# Hyaluronic Acid Oligosaccharides Suppress TLR3-Dependent Cytokine Expression in a TLR4-Dependent Manner

**DOI:** 10.1371/journal.pone.0072421

**Published:** 2013-08-23

**Authors:** Margaret Y. Kim, Jun Muto, Richard L. Gallo

**Affiliations:** Division of Dermatology, University of California San Diego and VA San Diego Health Care System, San Diego, California, United States of America; University of North Dakota, United States of America

## Abstract

The release of endogenous molecules from the skin after injury has been proposed to influence inflammation. Recent studies have found that pro-inflammatory signals can be generated by damaged endogenous self-RNA, and this event is detected by TLR3. Conversely, release of endogenous fragments of hyaluronic acid (HA) after injury has been proposed to inhibit LPS induced inflammation driven by TLR4. In this study we investigated if HA oligomers could also influence inflammation mediated by TLR3. A tetramer form of HA (oligo-HA) was added to MH-S cells (mouse alveolar macrophage cell line) that were then activated by poly(I:C). ELISA analysis of culture supernatants showed that the presence of oligo-HA suppressed the poly(I:C) induced release of IL-6 and TNFα. IL-6 mRNA expression was also suppressed as measured by quantitative RT-PCR. To determine the mechanism of action for oligo-HA to inhibit poly(I:C), macrophages derived from wild-type (WT), *Tlr2−/−* or *Tlr4−/−* mice were treated with oligo-HA and poly(I:C). Similar to WT cells, *Tlr2−/−* macrophages were inhibited by oligo-HA and retained suppression of cytokine release. In contrast, *Tlr4−/−* macrophages lost the capacity to be suppressed by oligo-HA. An increase in *Traf1* (TLR negative regulator) mRNA was observed after oligo-HA treatment of WT but not in *Tlr4−/−* macrophages, and oligo-HA did not suppress cytokine responsiveness in *Traf1−/−* macrophages. These results show that oligo-HA acts through TLR4 and TRAF1 to inhibit TLR3-dependent inflammation. This observation illustrates the complex immunomodulatory action of endogenous products released after injury.

## Introduction

Immediate defense against pathogen invasion depends on pattern recognition receptors (PRRs) such as Toll-like receptors (TLRs) to identify the presence of danger from microbial invasion [1]. Previously, this system was thought to function exclusively to recognize pathogens. However, this paradigm has shifted with increased appreciation that TLRs recognize not only highly-conserved molecules present on microbes (also known as “pathogen associated molecular patterns” (PAMPs)) but also responds to self molecules produced after cell damage or death (also known as damage-associated molecular patterns (DAMPs)) [2]. DAMPs include a variety of different molecules present in the host including ATP, heparan sulfate, High mobility group protein B1 (HMGB1), S100 proteins and hyaluronan [2]. Recently, release of RNA has also been shown to trigger inflammation after injury and is dependent on recognition by TLR3 [3], [4]. It is currently unclear how these multiple molecules interact with microbial products present in the complex environment of the wound, but several studies have suggested that the interplay between host and microbial-derived molecules serves to modulate the repair process and influences wound repair [5].

The interconnected associations between PAMPs and DAMPs can be seen by previous work done with hyaluronan (HA) [6]. HA is produced at the cell surface and released into the extracellular matrix as a high molecular weight linear glycosaminoglycan composed of repeating disaccharide subunits of D-glucuronic acid and N-acetyl glucosamine. The function of HA was initially thought to be mainly structural, but this paradigm changed when receptors to HA and its ability to regulate multiple functions were found. HA regulation is now recognized to be important both early in development and in various biological processes such as wound healing [7], [8]. Further studies have deduced that the function of HA is dependent on its size. HA of mixed sizes of less than 500 kDa induced inflammatory responses, whereas large molecular weight HA mixtures are anti-inflammatory and anti-angiogenic [7]. HA binds several different proteins that are able to influence immune function such as CD44, RHAMM (receptor for HA-mediated motility), TLR2 and TLR4 [9]–[11]. CD44 is a widely expressed HA receptor and plays a role in a variety of different biological mechanisms [12]. HA binding to CD44 has been found during development [13], inflammation and tumor growth [14]. Additionally, HA has been shown to engage a unique receptor complex of CD44 and TLR4 and induce cytokine release [15]. Clinically, large molecular weight HA has been successfully used as a clinical treatment for arthritis [16].

The potential for TLR cross-talk exists in a scenario of injury and infection where exposure to microbial products increases simultaneously with the generation of host-derived molecules. It has been previously reported that a mixture of HA of different sizes has the ability to alter TLR4 signaling and reduce inflammatory responses *in vitro* and *in vivo* in a CD44-dependent manner [6], [17]. Injection with the microbial derived TLR4 agonist LPS (lipopolysaccharide) by itself will induce a septic response in mice that is decreased when mice are also exposed to HA [6]. Poly(I:C) (polyinosinic:polycytidylic acid), a TLR3 agonist, can be used to mimic the response to endogenous RNA release after tissue necrosis and will also induce a septic response when injected into mice [18]. The influence of HA on this process is currently unknown. In this study we investigated if HA modifies the response to TLR3 mediated stimulation. Our results using a pure tetramer of HA (oligo-HA) demonstrate the capacity of HA fragments to modulate poly(I:C) induced cytokine responses *in vivo* and *in vitro*.

## Results

### Small HA Tetramers (oligo-HA) Suppress Poly(I:C)-induced Cytokine Secretion in Mice

**Figure 1:**
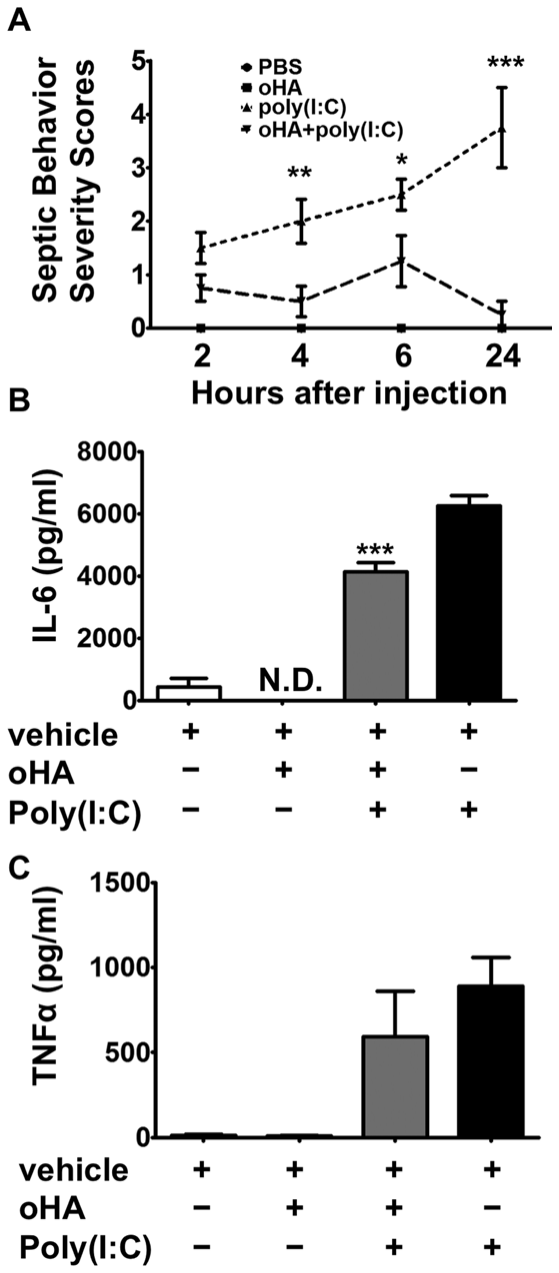
Oligo-HA protects mice from poly(I:C) induced shock response and inhibits cytokine release. Mice were injected i.p. with vehicle (PBS), oligo-HA (100 µg/mouse), oligo-HA and poly(I:C) (100 µg/mouse), or poly(I:C) alone. (A) Septic behavior severity scales were scored as described in Materials and Methods. Serum IL-6 (B) or TNFα (C) was measured 6 h after injection by ELISA. Data are means ± S.E.M. and are representative of three independent experiments with n = 4 per group. *: p<0.05, **: p<0.01, ***: p<0.001, N.D.: not detected.

A mouse model of poly(I:C)-induced shock was used to investigate the effects of tetramer oligosaccharides of HA on poly(I:C) signaling *in vivo*. Four groups of C57BL/6 wild-type (WT) mice were injected with PBS, oligo-HA (100 µg/mouse), oligo-HA and poly(I:C) (100 µg/mouse) or poly(I:C) alone, respectively. The mice were observed for septic behavioral responses: sunken eyes, raised/ruffled fur, shivering and slowed movement. Septic behavior response was monitored at 2, 4, 6, and 24 hours after injection. PBS and oligo-HA injected mice showed no increase in septic behavior but mice injected with poly(I:C) alone displayed severe septic responses (Figure 1A). However, if oligo-HA and poly(I:C) were injected together a major improvement in the septic response was seen at 4, 6 hours and 24 hours after injection (Figure 1A). This group of oligo-HA and ploy(I:C) treated mice had significantly reduced serum IL-6 (Figure 1B) compared to mice treated with poly(I:C) alone. Serum TNFα (Figure 1C) was not significantly decreased at this time point. These results suggested that oligo-HA can protect mice from poly(I:C)-induced experimental septic responses.

### Hyaluronan Oligosaccharide Treatment Alters Macrophage Inflammatory Responses to Poly(I:C)

**Figure 2:**
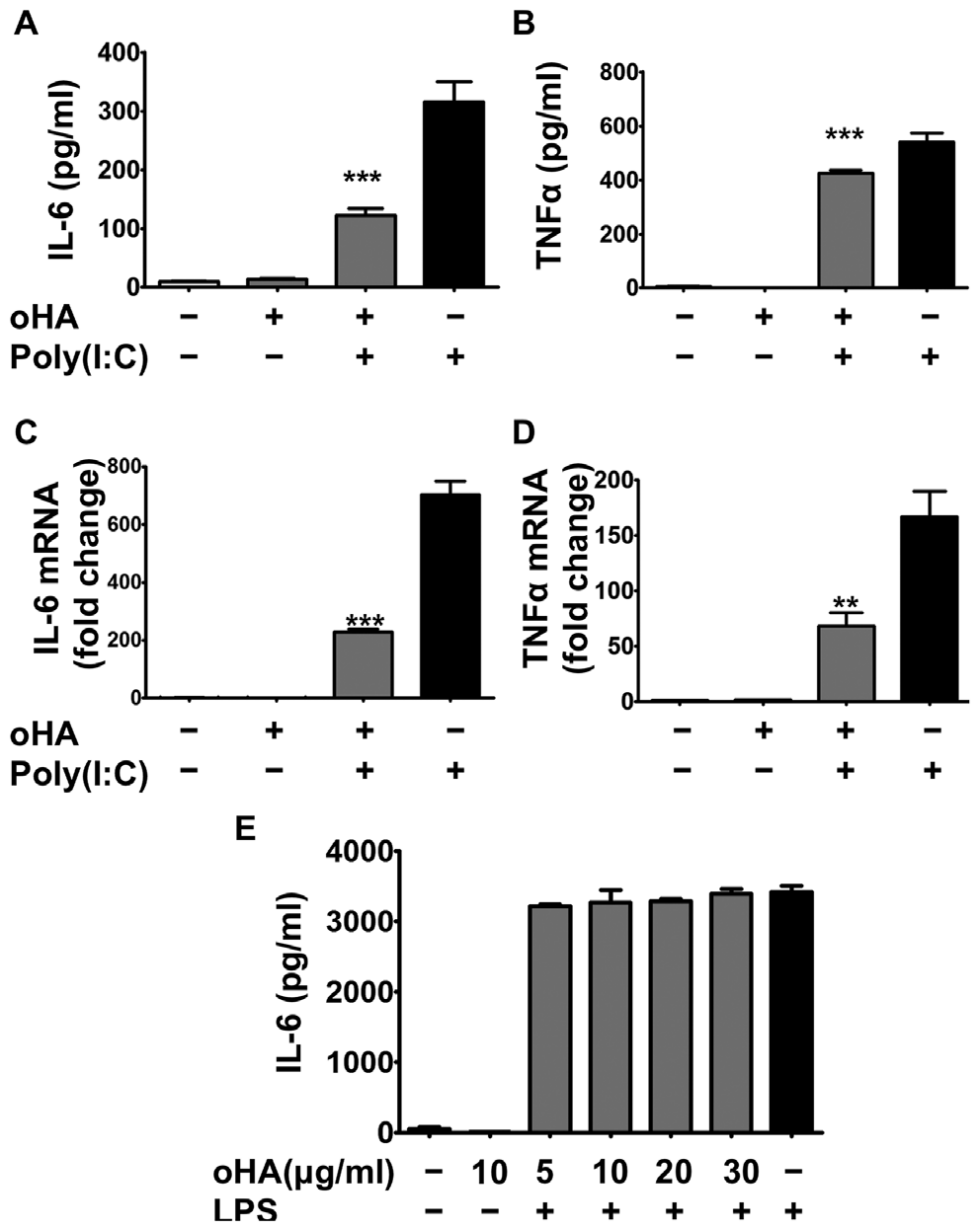
Oligo-HA suppresses poly(I:C) induced, but not LPS-induced cytokine release in MH-S cells. MH-S cells were treated with vehicle (PBS), oligo-HA (10 µg/ml), oligo-HA+poly(I:C) (10 µg/ml), or poly(I:C) alone for 24 h. IL-6 (A) and TNFα (B) release in the cultured media was measured by ELISA. mRNA abundance for IL-6 (C) and TNFα (D) was assessed 4 h after treatment by quantitative RT-PCR. Quantitative RT-PCR data are shown as relative expression as compared with vehicle-treated macrophages. (E) MH-S cells were treated with 100 ng/ml LPS in the presence or absence of oligo-HA for 24 hours. IL-6 release in the cultured media was measured by ELISA. Data are representative of five independent experiments with n = 3 per group and are means ± S.E.M. ***: p<0.001. **: p<0.01.

To determine how oligo-HA can suppress cytokine expression, an alveolar macrophage mouse cell line (MH-S cells) was treated with poly(I:C) and/or oligo-HA. IL-6 and TNFα release was suppressed in macrophages co-stimulated with oligo-HA and poly(I:C) compared to the cells treated with poly(I:C) alone (Figure 2A, B). A similar inhibition of mRNA abundance was also observed for IL-6 (Figure 2C) and TNFα (Figure 2D). In contrast to the suppression observed by oligo-HA on Poly(I:C), cells stimulated with LPS (100 ng/ml) were not inhibited by the addition of several concentrations of oligoHA (Figure 2E).

### TLR4 is Required for Oligo-HA to Suppress Cytokine Expression

**Figure 3:**
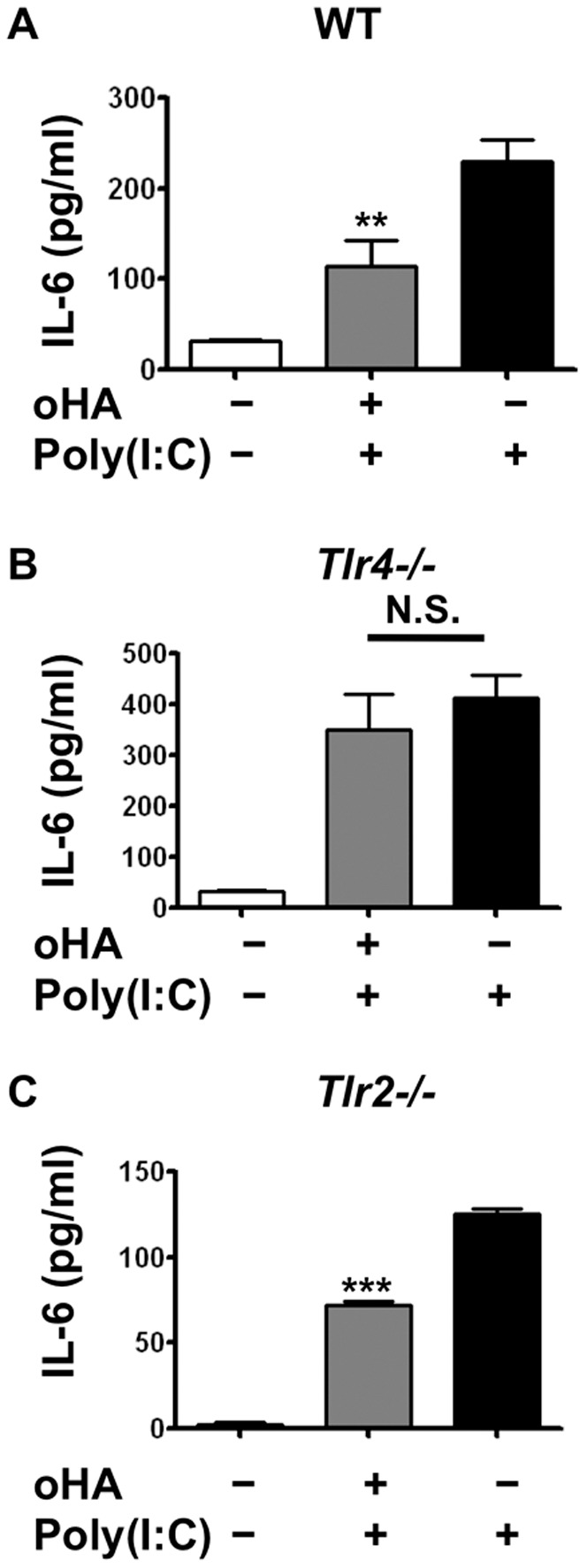
TLR4 is required for oligo-HA to suppress cytokine secretion and mRNA expression in peritoneal macrophages. WT (A), *Tlr4−/−* (B) and *Tlr2−/−* (C) peritoneal macrophages were treated with 10 µg/ml oligo-HA+10 µg/ml poly(I:C), poly(I:C) alone or vehicle (PBS) for 24 h. IL-6 release in the cultured media was measured by ELISA. Data are means ± S.E.M. and are representative of three independent experiments with n = 3 per group. ***: p<0.001, **: p<0.01, N.S.: not significant.

Previous findings have suggested that HA can be recognized by TLR2 or TLR4 [7]. To further elucidate the mechanism by which oligo-HA exerted inhibitory effects on cytokine production by macrophages, peritoneal macrophages were isolated from WT, *Tlr4−/−* and *Tlr2−/−* mice and treated with or without oligo-HA to observe the differences in poly(I:C)-induced cytokine expression. Similar to results obtained with MH-S cell line, oligo-HA treatment of macrophages from WT mice inhibited IL-6 release stimulated by poly(I:C) (Figure 3A). However, oligo-HA treatment of macrophages from *Tlr4−/−* mice did not exhibit this same suppression (Figure 3B). In contrast, oligo-HA treatment of macrophages from *Tlr2−/−* mice showed suppression similar to macrophages from WT mice (Figure 3C). These results indicated that the suppression of poly(I:C)-induced cytokine release by oligo-HA was dependent on TLR4, but not TLR2.

### Inhibition of Inflammatory Response to Poly(I:C) by Oligo-HA is Dependent on TRAF1, a Negative Regulator of TLR Signaling

**Figure 4:**
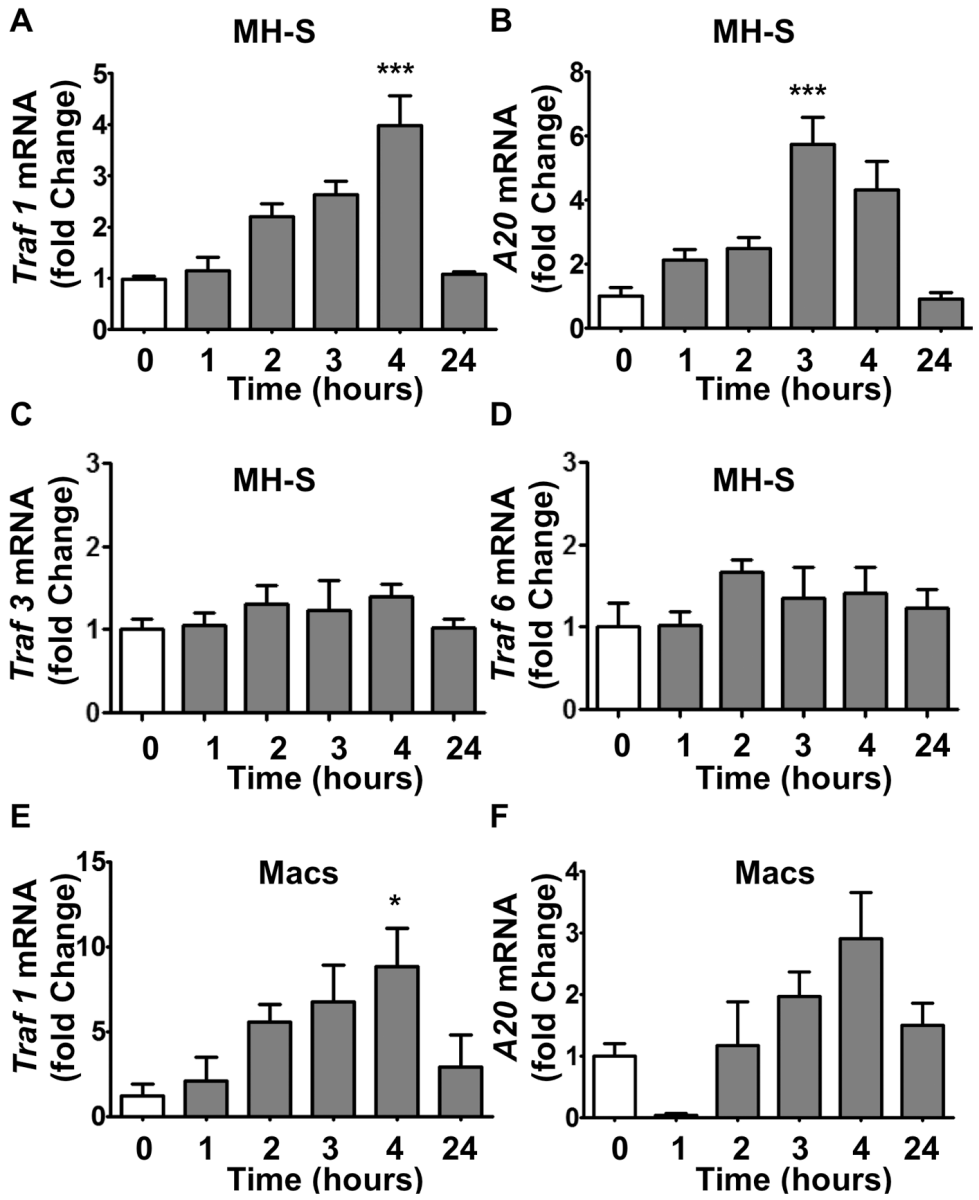
Oligo-HA induces inhibitors of TLR3 signaling via TLR4. MH-S cells (A, B, C, D) and peritoneal macrophages (Macs) from C57Bl/6J mice (E, F) were treated with 10 µg/ml oligo-HA or vehicle control for indicated time points. *Traf1*, *Traf3*, *Traf6* and *A20* mRNA expression was assessed by quantitative RT-PCR. Quantitative RT-PCR data are shown as relative expression compared to vehicle-treated macrophages. Data are means ± S.E.M. and representative of three independent experiments with n = 3. *: p<0.05; ***: p<0.001.

To explore how oligo-HA might act to inhibit TLR signaling we next investigated the effects of oligo-HA on the expression of *A20* and *Traf1*, both known regulators of TLR function. Oligo-HA up-regulated both *Traf1* (Figure 4A) and *A20* (Figure 4B) mRNA expression in MH-S cells, but not *Traf3* (Figure 4C) and *Traf6* (Figure 4D). Peritoneal macrophages from WT mice also showed an induction of *Traf1* (Figure 4E) and *A20* (Figure 4F) after oligo-HA treatment. These results indicated that oligo-HA treatment could induce these negative regulators of TLR3 in macrophages.

**Figure 5:**
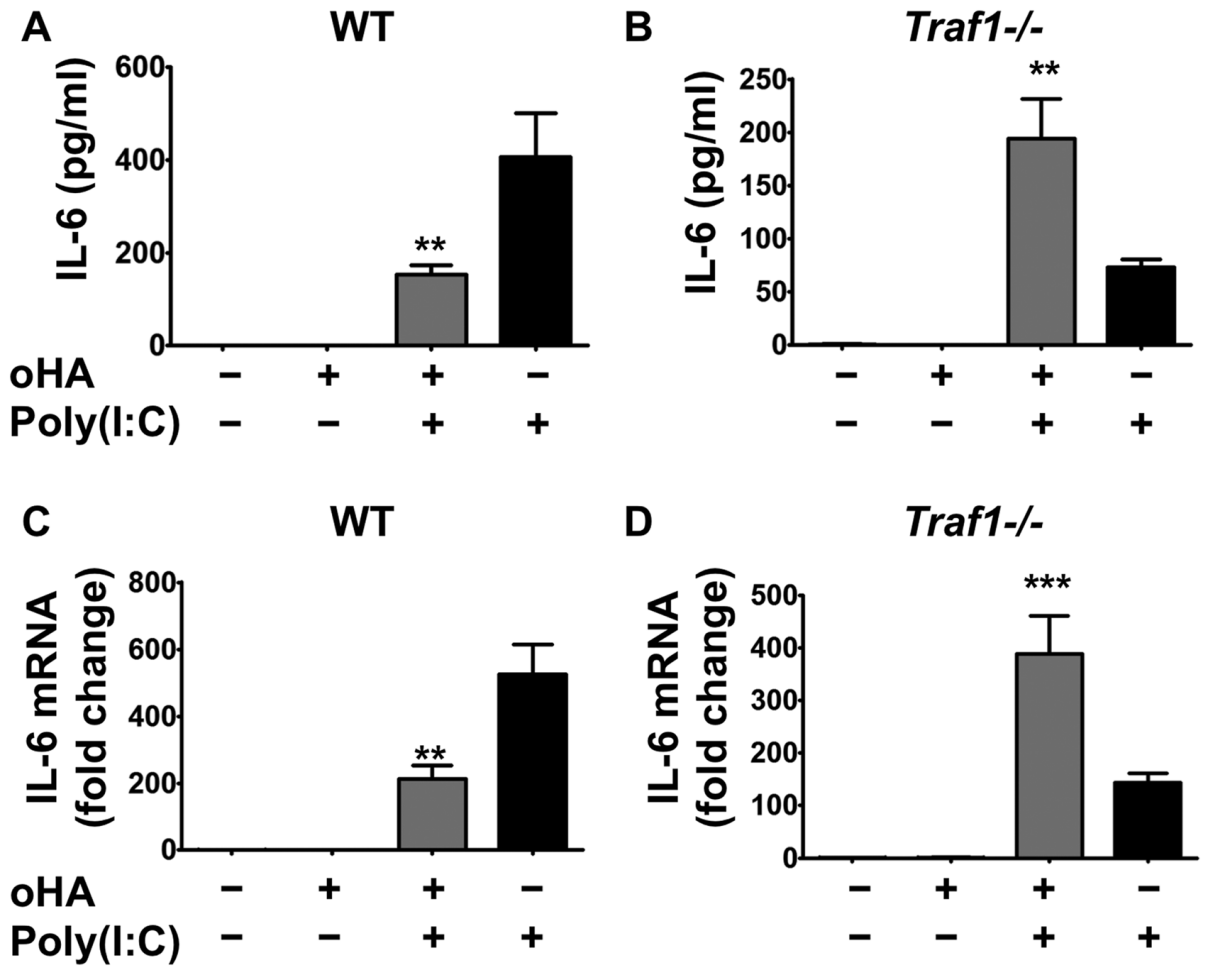
TRAF1 is necessary for oligo-HA to suppress IL-6 secretion and mRNA expression in peritoneal macrophages. Peritoneal macrophages from WT (A, C) and *Traf1−/−* mice (B, D) were treated with oligo-HA and/or poly(I:C) for 24 h. IL-6 release in cultured media was measured by ELISA (A, B). mRNA abundance for IL-6 was assessed by quantitative RT-PCR 4 h after treatment (C, D). Quantitative RT-PCR data are presented as normalized data against vehicle-treated macrophages. Data are presented as means ± S.E.M. and are representative of three independent experiments with n = 3. **: p<0.01. ***: p<0.001.

**Figure 6:**
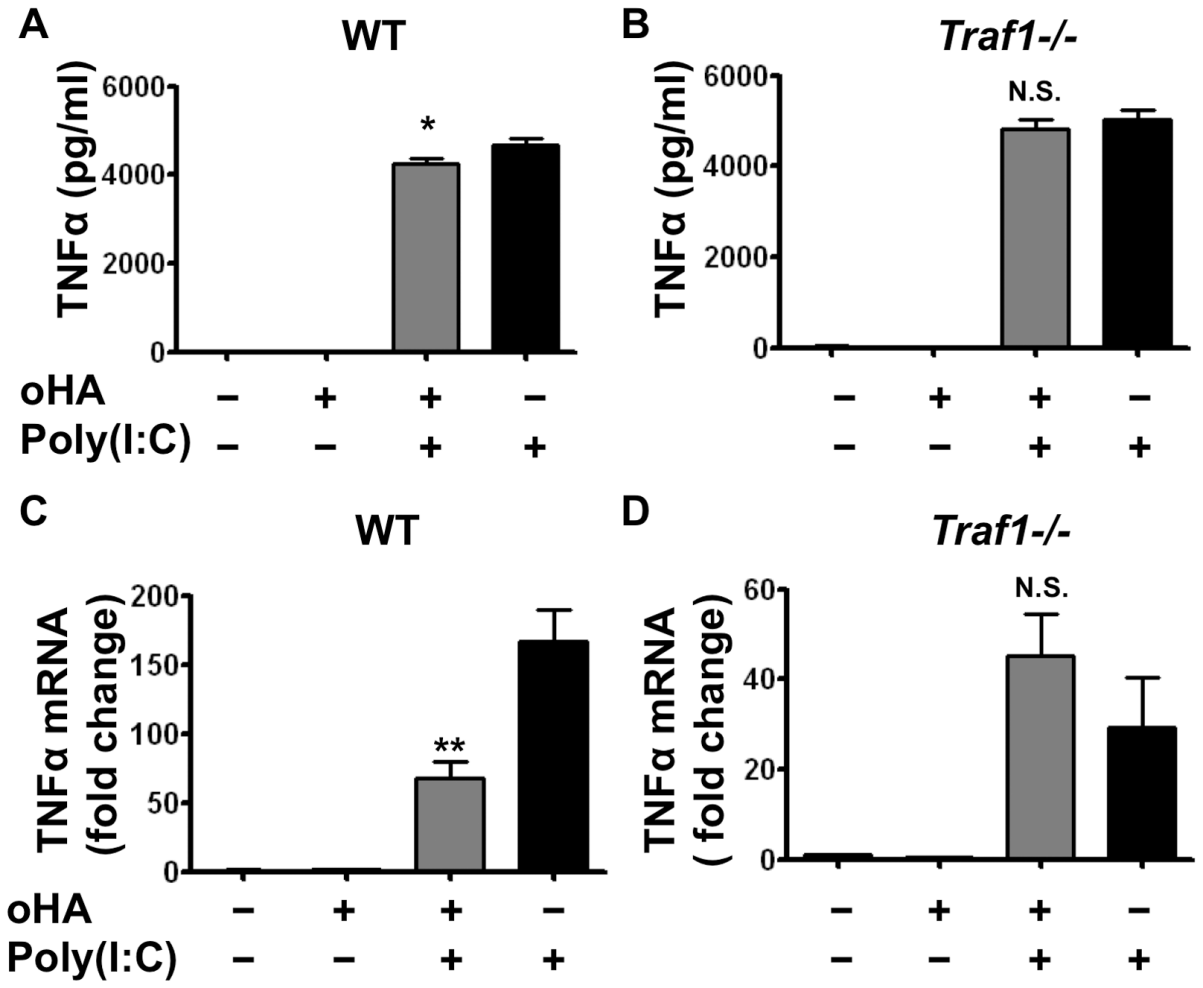
TRAF1 is necessary for oligo-HA to suppress TNFα secretion and mRNA expression in peritoneal macrophages. Peritoneal macrophages from WT (A, C) and *Traf1−/−* mice (B, D) were treated with oligo-HA and/or poly(I:C) for 24 h. TNFα release in cultured media was measured by ELISA (A, B). mRNA abundance for TNFα was assessed by quantitative RT-PCR 4 h after treatment (C, D). Quantitative RT-PCR data are presented as normalized data against vehicle-treated macrophages. Data are presented as means ± S.E.M. and are representative of three independent experiments with n = 3. *: p<0.05. **: p<0.01, N.S.: not significant.

Next, to determine the dependence on TRAF1 for oligo-HA-mediated cytokine suppression of TLR3 signaling, we treated macrophages from WT and *Traf1−/−* mice with poly(I:C) and/or oligo-HA as in Figure 2. Oligo-HA treatment of WT macrophages inhibited poly(I:C) induction of IL-6 protein (Figure 5A). In contrast, *Traf1−/−* macrophages stimulated with poly(I:C) were not able to be inhibited by oligo-HA (Figure 5B). This was also confirmed at the mRNA level, where poly(I:C) induction of IL-6 mRNA was suppressed in macrophages from WT (Figure 5C), but not *Traf1−/−* mice (Figure 5D). Interestingly, we also found the levels of IL-6 mRNA and protein in the poly(I:C)-alone treated macrophages from *Traf1−/−* mice were lower than those in WT mice. In contrast to WT mice where oligo-HA treatment inhibited the expression of IL-6 relative to poly(I:C) alone, *Traf1−/−* mice responded to oligo-HA treatment with improved sensitivity. A similar trend was observed for TNFα mRNA and protein under these conditions (Figure 6A–D). These data suggest that TRAF1 is involved in oligo-HA-mediated suppression of poly(I:C), but in the absence of the suppressive action of TRAF1, oligo-HA recognition may enhance some cytokine responses.

## Discussion

Prior studies have shown that endogenous large molecular weight HA is degraded *in vivo* and that mixed preparations of degraded HA had potential for suppressing some inflammatory events in cultured cells and mice. Here we show for the first time that a small HA oligosaccharide is sufficient to induce responses of physiological relevance to RNA-induced inflammation, a finding of relevance to a wound repair environment. Our data show that inhibition of RNA-induced cytokine expression is dependent on both TLR4 and TRAF1 as the absence of either gene prevented the capacity of HA to reduce cytokine production. Therefore, these findings support the hypothesis that the degradation of HA in the ECM could play an important role in regulating host responses to wounding.

Intermediate sizes of HA (200–500 kDa) can induce inflammatory cytokines [7], and thus fragments of large molecular weight HA (>500 kDa) have been proposed to be a “danger signal” associated with sterile injury [19]. We used the tetramer form of HA to provide a specific assessment of small molecular weight HA in a setting of activation by double-stranded RNA. Interestingly, this size HA was potently able to inhibit poly(I:C). Previously, mixed size preparations of HA were found to dampen LPS signaling [6]. Further work is necessary to specifically define which size of HA fragments function to inhibit LPS activation and to understand why oligo-HA is dependent on TLR4 but does not inhibit LPS activation through TLR4.

Precise regulation of the activation of TLRs is necessary to establish the appropriate balance between inflammation and repair. It is essential for the host to attenuate TLR signaling to avoid detrimental effects of excess inflammation responses. TLR3 signaling has only recently become of interest to wound healing and proposed as an endogenous sensor of injury [20], [21]. Several intracellular proteins have been identified as negative regulators of TLR signaling [22], [23], and our observations support the hypothesis that oligo-HA acts through TNF receptor-associated factor 1 (TRAF1), one of the negative regulators of the TRIF pathway downstream of TLR3 [23]. TRAF1 becomes active to inhibit TRIF after digestion to generate an N-terminal fragment that binds to the TIR-domain (Toll/interleukin-1 receptor) of TRIF [24]. Furthermore, the C-terminal product of TRAF1 has also been suggested to block IKK activation through physical interaction with IKK and inhibition of NF-κB activation [25]. It remains to be determined how HA acts to enable TRAF1 to inhibit TLR3, however other examples of induction of TRAF1 have been described including IL-1, TNFα, CD40 ligand and EBV infection [26], and microbial activation through Lipotechoic acid and TLR2 [3]. TRAF1 is highly inducible by NF-κB, which may explain why various stimuli that activate NF-κB also induce TRAF1 expression [27]. The physiological relevance of TRAF1 is supported by observations that polymorphism of genes involved in TNF-receptor signaling such as TRAF1 are associated with rheumatoid arthritis, mortality in sepsis and malignancy [28]–[30]. A20 is a more ubiquitous negative regulator and has the ability to deubiquitylate TRAF6, an important downstream molecule for TLR signaling [31]. We had previously shown that the expression of A20 can be induced by HA treatment in macrophages but it was unclear which specific size of HA has this effect [6]. In the present study HA was also seen to increase the expression of A20. However, its role in mediating the response to HA is not yet known since suppression of poly(I:C)-induced inflammation did not occur in macrophages from *Traf1−/−* mice. In contrast, we found that macrophages from *Traf1−/−* mice responded to oligo-HA in the opposite manner to WT macrophages. Rather than inhibiting IL-6 and TNFα expression, these macrophages showed increased sensitivity to poly(I:C). This response was observed in a background of lesser relative responsiveness to poly(I:C) alone in *Traf1−/−* macrophages. The underlying mechanism for this lower baseline sensitivity remains to be determined.

The ability of oligo-HA to induce the negative regulator TRAF1 and suppress cytokine induction has therapeutic implications, especially in relation to responses to traumatic injury. During wounding, fragments of HA reside locally where damage initially occurs and may limit inflammation. This may benefit the host to prevent excess inflammation that could result in local tissue damage. However, this effect must be strictly controlled since too little inflammation may also increase risk of infection and delay wound healing. Specifically, deletion of TLR3 signaling has been shown to be detrimental to host wound healing via impaired recruitment of myeloid cells [20].

Our observation that oligo-HA pretreatment protects mice from the poly(I:C)-induced experimental shock phenomenon and suppresses cytokine elevation in serum sheds new light on potential elements that regulate septic shock responses and may have important therapeutic implications. Our findings suggest that HA may be a natural regulator of septic shock responses since HA is an abundant glycosaminoglycan present in the extracellular matrix of many tissues and available for release in soluble form upon tissue injury [15]. A lack of appropriate capacity to activate this dampening system may predict adverse responses seen in some individuals following a wide variety of traumatic insults in several organs systems including skin and lung.

In summary, our data demonstrates that oligo-HA has the ability to alter the inflammatory response to poly(I:C) in a TLR4 dependent manner. The suppression is dependent on the induction of TRAF1, a negative regulator of TLR3. These results more clearly define the capacity of HA to alter inflammatory responses to multiple stimuli.

## Materials and Methods

### Ethics Statement

This study was carried out in strict accordance with the recommendations in the Guide for the Care and Use of Laboratory Animals of the National Institutes of Health (NIH). Experiments of using mice were performed at University of California, San Diego (UCSD). The UCSD ethics committee specifically approved this study under an approved Institutional Animal Care and Use Committee (IACUC) protocol (no. S09074).

### Reagents, Cell Lines, Mice

The mouse alveolar cell line MH-S was purchased from American Type Culture Collection (ATCC, catalog CRL-2019). Cells were maintained in RPMI1640 media supplemented with L-glutamine, 10% heat-inactivated fetal calf serum (FCS), penicillin/streptomycin (100 units/ml and 50 mg/ml, respectively), and 50 µM 2-mercaptoethanol. Tetrasaccharide-hyaluronan (endotoxin level less than 0.1 EU/mg oligosaccharide) was purchased from Hyalose, L.L.C. (Oklahoma City, OK). Poly(I:C) was obtained from Invivogen (Carlsbad, CA). LPS (from E. Coli K12D31m4) was obtained from LIST Biologics, Inc. (San Diego, CA). C57Bl/6J wild-type, *Tlr4*-deficient and *Traf1*-deficient mice were purchased from Jackson Laboratories (Bar Harbor, ME). Eight- to ten-week old/sex matched animals were used for experiments. The animal experiments were approved by UCSD’s institutional animal care and use committees and were conducted in accordance with the guideline for the care and use of laboratory animals.

### Experimental Shock and Mouse Behavioral Studies

Age and sex-matched female wild-type mice were used. Mice were injected with 100 µg/mouse HA or PBS intraperitoneally (i.p.) as a pretreatment. One hour after pretreatment, mice were then injected with 100 µg/mouse poly(I:C) or PBS i.p. to induce experimental septic symptoms. Mice were then monitored over time for changes in behavior as previously described [6].

### Peritoneal Macrophage Isolation

Age and sex-matched WT, *Tlr2−/−*, *Tlr4−/−*, and *Traf1−/−* were injected i.p. with 3% thioglycolate (Sigma-Aldrich, St. Louis, MO). After 72 hours, peritoneal macrophages were collected by peritoneal lavage and were plated onto 24-well tissue culture plates at 3×10^7^ cells/well in RPMI1640. The adherent peritoneal macrophages were washed with PBS and cultured in 10% heat-inactivated FCS/RPMI1640 and rested for 24 hours. After recovering, cells were stimulated and supernatants and RNA were collected.

### *In vitro* Cell Stimulation and Sample Collections

Macrophages were grown to confluence in 24-well flat bottom plates (Corning Incorporated Life Sciences, Lowell, MA). For experiments, media was removed from cells and replaced with media containing vehicle, HA, poly(I:C) or both at the indicated concentrations. All stimulations were done in low serum media (1%). After stimulation, cells were allowed to incubate for indicated time, then media were collected and stored at −20°C until analysis. RNA was extracted from adherent cells after supernatant collection using TRIzol reagent (Invitrogen, Carlsbad, CA) and stored at −80°C. For the experiments of LPS induced cytokine release, cells were treated with 100 ng/ml LPS in the presence or absence of HA for 24 hours and media were collected and stored until analysis.

### Quantitative RT-PCR

Quantitative RT-PCR was used to determine the induction of *Il-6*, *Tnfα*, *Traf1*, *Traf3*, *Traf6*, *A20* mRNA after stimulation with oligo-HA and poly(I:C). cDNA was synthesized from RNA by iScript cDNA synthesis kit (BioRad, Hercules, CA) as described by the manufacture’s protocol. TaqMan™ Gene Expression Assays (Applied Biosystems, Foster City, CA) were used to analyze mRNA expressions. *Gapdh* mRNA was used as an internal control to validate mRNA for each sample. *Il-6*, *Tnfα*, *Traf1*, *Traf3*, *Traf6* and *A20* mRNA were calculated as relative expression to *Gapdh* mRNA and all data are presented as normalized data against each control (mean of non-stimulated cells) [19].

### Analysis of Supernatants and Sera by ELISA

Supernatant or mouse serum cytokine concentrations were analyzed by species-specific IL-6 and TNFα through ELISA (R&D systems, Minneapolis, MN).

### Statistical Analysis

One-way ANOVA with Tukey post-tests or two-way ANOVA with Bonferroni post-tests were used to determine significance in the experiments, analyzed through Graphpad Prism 5 (GraphPad Software Inc., La Jolla, CA).
